# Risk Factors for Neurocognitive Functioning in Children with Autosomal Recessive Polycystic Kidney Disease

**DOI:** 10.3389/fped.2017.00107

**Published:** 2017-05-15

**Authors:** Stephen R. Hooper

**Affiliations:** ^1^Department of Allied Health Sciences, School of Medicine, University of North Carolina at Chapel Hill, Chapel Hill, NC, USA

**Keywords:** autosomal recessive polycystic kidney disease, pediatric autosomal recessive polycystic kidney disease, neurocognition in autosomal recessive polycystic kidney disease, risk factors for neurocognitive dysfunction in autosomal recessive polycystic kidney disease, polycystic kidney diseases

## Abstract

This mini review provides an overview of the issues and challenges inherent in autosomal recessive polycystic kidney disease (ARPKD), with a particular focus on the neurological factors and neurocognitive functioning of this population. ARPKD typically is discovered at the end of pregnancy or during the neonatal developmental period and occurs in approximately 1 in 20,000 live births. During the neonatal period, there is a relatively high risk of death, with many infants dying from respiratory failure. As the child ages, they experience progressive kidney disease and become increasingly vulnerable to liver disease, with many individuals eventually requiring dual organ transplants. This mini review provides a brief description of ARPKD and describes the various factors that place children with ARPKD at risk for neurological and neuropsychological impairment (e.g., a genetic condition leading to chronic kidney disease and eventual transplant; difficult-to-treat hypertension; eventual liver disease; possible dual transplantation of the kidneys and liver; chronic lung disease), including that these factors are present during a critical period of brain development. Further, the mini review discusses the available studies that have addressed the neurocognitive functioning in children with ARPKD. This paper concludes by providing the key clinical and research challenges that face the field of pediatric nephrology with respect to the clinical and scientific study of the neurocognitive functioning of children with ARPKD. Selected directions are offered in both the clinical and research arenas for this multiorgan chronic condition.

## Introduction

Cystic kidney diseases represent a heterogeneous group of renal conditions characterized by tubular cysts, glomerular cysts, or dysplasia. Within this heterogenous group, polycystic kidney disease typically refers to two conditions: autosomal dominant polycystic kidney disease (ADPKD) and autosomal recessive polycystic kidney disease (ARPKD), that are genetically distinct (1, 2). In most instances, these two conditions manifest in different developmental epochs, with the ARPKD being associated with younger children and ADPKD being associated with adults; however, given their genetic basis, both conditions can appear at any developmental time period. For this special issue, a key focus has been placed on ARPKD and its various characteristics. The purpose of this paper is to address the state of the literature regarding the neurocognitive functions and associated neurodevelopmental issues and challenges presented by ARPKD.

## Epidemiology and Early Developmental Course of ARPKD

Autosomal recessive polycystic kidney disease occurs at a lower frequency than its genetic counterpart, ADPKD, with incidence being about 1:20,000 live births (3), with rates ranging from 1:6,000 to 1:55,000 (1, 4). Many affected infants may die during the neonatal period secondary to respiratory failure, suggesting that these incidence rates actually may underestimate the true number of individuals with ARPKD. In the past, a diagnosis of ARPKD was believed to be a fatal condition at birth, with infant death now estimated to be at approximately 23–30%. Those surviving the neonatal period typically will have a 10-year survival rate of around 80% (5), with the risk of death secondary to liver disease increasing with age (6). Concomitantly, renal disease also advances, albeit slowly (7), with about 30% reaching end-stage renal disease before the age of 10 years, and another 30% during adolescence (8). Alarmingly, emergent data from individuals with ARPKD, who received a kidney transplant, suggest that approximately 80% subsequently developed liver disease, which not only complicated the kidney transplant but also increased the risk of death (6). There are no differences in incidence rates across gender or race/ethnicities.

Autosomal recessive polycystic kidney disease occurs from mutations in one gene, the Polycystic Kidney and Hepatic Disease 1 gene (PKHD1). The PKHD1 gene encodes the fibrocystin protein whose functions remain unclear at this time. In most cases, this condition is uncovered during the end of pregnancy or during the neonatal period (9), with the infant manifesting significantly enlarged kidneys, progressive renal insufficiency, and arterial hypertension (2, 3), the latter being present in nearly 80% of infants. Further, many infants will show pulmonary insufficiency that can result in death in about one-third of afflicted infants. For nearly all infants, the early clinical presentation is characterized by kidney dysfunction and, as the child ages, disruptions of liver function (10). In general, this condition will manifest very early in development; however, a small subset of cases will manifest symptoms during later development, with symptoms being dominated by liver complications (11).

## Risk Factors for Neurocognitive Dysfunction in ARPKD

There are multiple neurological conditions that can be associated with renal disease. These include central nervous system conditions such as uremic encephalopathy syndrome, seizures, movement disorders, and strokes—which can occur at alarming rates, sleep disorders, and a variety of peripheral nervous system conditions (e.g., polyneuropathy) (12). There are also neurological symptoms that can be associated with dialysis procedures (13) and transplant (14). All of these neurological conditions can and will contribute to neurocognitive deficits and dysfunction in children with ARPKD. Additionally, children and adolescents with ARPKD have a number of other vulnerabilities that can contribute to the manifestation of various neurodevelopmental challenges, particularly cognitive difficulties.

### Chronic Kidney Disease (CKD)

The mere fact that individuals evidence CKD places them at varying degrees of risk for neurocognitive and social–behavioral challenges throughout their life span (15). Approximately 40% of children and adolescents with mild-to-moderate CKD have been shown to exhibit some form of neurocognitive dysfunction, perhaps secondary to the associated cardiovascular risk and other factors associated with CKD (16). Although there are no clear data suggesting when children with ARPKD will progress to end-stage renal disease, or the factors that contribute to this progression in this population, many children with ARPKD ultimately may need renal replacement therapy. These therapies, particularly dialysis, can also contribute to the manifestation of neurocognitive difficulties. For example, for the youngest affected children, progression can be rapid and they may require dialysis early in life. It has been estimated that approximately 25% of very young children with severe renal difficulties will have significant developmental delays, and very young infants and toddlers with ARPKD on dialysis likely are similarly affected (17).

### Hypertension

In conjunction with CKD, children with ARPKD also are at risk for hypertension. Although there are little extant data specific to children with ARPKD, there is growing evidence that primary hypertension (18–22), and even variability in blood pressure (23), are associated with altered neurocognition in children. The potential cause is unclear, though investigators have posited that chronic hypertension may result in small vessel damage, which, in turn, leads to reduced or impaired regulation of cerebral blood flow (24) that can impact brain development over time. In particular, the development of executive functioning, a protracted process that extends into young adulthood, may be particularly vulnerable to alterations in the capacity of vessels to dilate in response to local or regional changes in neuronal activity (21). More specifically, elevated blood pressure and hypertension in children and adolescents have been associated with lower scores on performance-based measures of working memory, non-verbal reasoning, and aspects of academic achievement compared to individuals with normal pressure (22). Importantly, most documented differences have occurred within the normal range of functioning, and there is preliminary evidence that executive functioning may improve with antihypertensive treatment (25). How these findings manifest in children with ARPKD remains to be determined, but the presence of early and significant hypertension in ARPKD put these children at risk for neurocognitive disruption.

### Hepatic Fibrosis

Another source of morbidity in children with ARPKD is hepatic fibrosis. With respect to the neurocognitive effects of liver disease, there have been several case reports of hepatic encephalopathy in individuals with ARPKD who have undergone portosystemic shunting (PSS) to relieve severe portal hypertension in patients with isolated congenital hepatic fibrosis (26). In individuals with ARPKD and with renal dysfunction, hyperammonemia after PSS may exceed the capacity for renal ammonia disposal, resulting in hepatic encephalopathy in ARPKD patients with PSS on chronic hemodialysis (5), in the immediate postrenal transplant period (6) and following bilateral nephrectomy (27). While these cases illustrate the potential for overt encephalopathy in ARPKD patients with PSS and advanced CKD, it is unknown whether patients with milder CKD could have subtle neurocognitive deficits following PSS. Further studies are therefore needed to detect whether “minimal hepatic encephalopathy” (28) could occur in this population. For children with ARPKD needing a liver transplant, findings from pediatric liver transplantation in other non-ARPKD-specific populations suggest additional potential difficulties including lower quality of life (29, 30), poorer family functioning (31), lower self-perceptions of physical attractiveness, and depressed mood (32). In fact, these cognitive and psychosocial difficulties do not seem to improve following transplantation over the short-term or the long-term (33), thus raising another potential concern for neurodevelopmental difficulties in children with ARPKD and liver involvement.

### Chronic Lung Disease

Finally, a third source of morbidity for children with ARPKD is chronic lung disease, particularly during the neonatal period and in those infants who are born prematurely. As noted earlier, many affected infants may die during the neonatal period secondary to respiratory failure, but many infants survive only to experience bronchopulmonary dysplasia, or chronic lung disease. Chronic lung disease is an inflammatory disorder that tends to manifest in the first few weeks of life, with about 40% of infants with ARPKD requiring mechanical ventilation and pharmacological treatments for the lung disease (9). For premature infants with chronic lung disease who survive their first year of life, there may be downstream neurocognitive deficits that comprised developmental delays noted at 2 years of age (34) as well as later deficits in IQ, gross motor functions, visual-spatial abilities, and core reading and math skills in middle childhood (35, 36). Consequently, the manifestation of chronic lung disease in infants with ARPKD creates yet another risk factor for neurodevelopmental difficulties as the child advances in age.

## Neurocognitive Findings in ARPKD

Given the neonatal risks that many individuals with ARPKD experience, the subsequent kidney function deterioration, hypertension, and liver disease, along with the associated treatments necessary to address these potential conditions, it would appear that all individuals with ARPKD are at significant risk for neurocognitive impairments—even with relatively successful treatments of these conditions. When these medical conditions are overlaid on the observations that these challenges are occurring during critical periods of early brain development, the risk for neurocognitive dysfunctions is heightened to a greater degree. Despite these significant risk factors for neurocognitive outcomes, there is a paucity of literature addressing neurocognition in children with ARPKD. There have been several case series presentations describing children with ARPKD (37, 38); however, despite what appears to be significant risk for neurocognitive impairment in this population, there are virtually no empirically based studies examining the neurocognitive functioning in children with ARPKD.

To our knowledge, there has been only one systematic investigation of neurocognition in children with ARPKD (39). This study examined results of comprehensive neurocognitive assessments in children with ARPKD in the chronic kidney disease in children (CKiD) cohort study, a multicenter investigation of children with mild-to-moderate CKD. To look for disease-specific neurocognitive effects of ARPKD, children with ARPKD were compared to a control group of children with renal aplasia/hypoplasia/dysplasia who were matched based on kidney function, age at study entry, and age at diagnosis. In all domains evaluated, which included intellectual functioning, academic achievement, attention regulation, executive functioning, and behavior, children with ARPKD performed comparably to the control group in this cohort. Both groups had scores within the average range in all domains, but a larger than expected proportion of children in both groups had scores in the “at-risk” range (i.e., worse than 1 SD below the mean) in some domains. These findings were consistent with prior studies of neurocognition in children with mild-to-moderate CKD (16). While these results are somewhat reassuring, they may not be generalizable to children with more severe manifestations of ARPKD given that the CKiD study only included children with mild-to-moderate CKD. Further studies examining the neurocognitive and social–behavioral functioning in children with ARPKD are sorely needed, with particular attention being devoted to improving our knowledge of the genotype–phenotype linkages for this single gene disorder.

## Challenges and Future Directions for ARPKD

### Clinical Best Practices

The meager literature suggests that children with ARPKD may not manifest neurocognitive difficulties; however, in addition to the limitations of the available literature and the medical risk factors present in all of these individuals, it is likely that children with ARPKD are at elevated risk for neuropsychological difficulties. Yet, the extant literature provides scant information to explicitly guide clinical practice with this population. Given the complexity of the disease and the involvement of multiple providers, it will be critical for the clinical care team to review a child’s neuropsychological status on a systematic basis. Both structured (e.g., questionnaire) and unstructured (e.g., clinical interview) data from multiple sources (child, caregivers, and teachers) are likely to provide sufficient data to alert the medical team to cognitive, behavioral, or learning difficulties that merit further assessment. Comprehensive neuropsychological assessment may be indicated for those children who show signs of learning and attention problems and for those who manifest behavioral or emotional difficulties, the latter areas being particularly understudied in children with CKD and ARPKD more specifically. Given the at-risk status of this population, obtaining a baseline assessment for children whose neurocognitive and psychosocial functioning appears relatively unaffected also will provide comparative data for when the child’s medical status and overall functioning begins to deteriorate. In addition to assisting in tracking developmental trajectories of various neurodevelopmental functions, such information is critical to the formation of developmentally appropriate intervention plans. These recommendations have been detailed via consensus expert recommendations provided by Guay-Woodford and colleagues (40).

Regardless of the assessment approach, an interdisciplinary team model is suggested (40–42). Ideally, the interdisciplinary team would include a consulting psychologist or neuropsychologist with specialized knowledge of CKD as well as the associated comorbid conditions (e.g., hypertension, liver disease), and the team would engage in systematic developmental surveillance, perhaps via brief annual or biannual screenings, to track children’s cognitive, social, and behavioral functioning over time and/or in tandem with disease progression. Although the timing of a comprehensive evaluation will vary depending on an individual child’s risk factors, it is suggested that children with ARPKD who are showing neurocognitive or social–emotional problems and/or who have increasing medical risk secondary to the presence of comorbid conditions, receive more frequent evaluations, perhaps at key developmental transition points (i.e., before school entry, prior to middle school, transition to high school, etc.) so that developmentally appropriate interventions could be implemented. This interdisciplinary team approach has been suggested for other pediatric conditions (e.g., feeding disorders) (43) and its application to children with kidney disease should be explored.

### Research

As can be seen from the above overview, there is a paucity of empirical findings pertaining to the neurocognitive and social–emotional functioning of children with ARPKD. This is not an oversight by the field but, rather, a challenge to ascertain samples large enough to study. Indeed, additional case study and case series descriptions of the various neurocognitive and social–emotional manifestations of ARPKD would provide scientific discussion as to research directions for this population. This could be particularly important so as to describe functioning across different disease severity levels. Research on ARPKD is also complicated by the multiple avenues for morbidity that need to be concurrently addressed in any empirical study given the potential effects of these factors on children’s neurocognitive and psychosocial functioning.

Given the inherent overlap of these conditions with ARPKD, it may be useful for the field to capture such data from larger cohorts across CKD, liver disease, and hypertension populations. Consideration for the establishment of a national patient registry, perhaps via a large clinical research consortium, also may fuel ascertainment of patients for a myriad of scientific investigations, including the neurocognitive and social–emotional functioning of these children over the course of development. The ARegPKD, a European ARPKD Registry Study, has initiated such a project; however, it appears at this time that their rational, design, and study objectives do not include any formal measures of neurocognition, neurodevelopment, or neurological functions (44). These latter features are critical to providing a comprehensive genotype–phenotype description of this complex genetic condition. As such, it will be critical for the field of pediatric nephrology to continue to advance and refine the genotype–phenotype linkages so as to assist with the clinical developmental needs associated with this condition. This is particularly important given the early age of diagnosis for most of these cases (i.e., the importance of early intervention for the neurodevelopmental needs), the extended life expectancy that has been provided with ongoing medical advances (i.e., the increased challenges faced by older children, adolescents, and young adults with a multiorgan chronic illness), and the need for such assessment methods in future clinical trials to treat ARPKD.

## Conclusion

This mini review provides an overview of the issues and challenges inherent in ARPKD, with a particular focus on the neurocognitive functioning of this population. Despite the relative dearth of literature on the neurodevelopmental functioning and development of children with ARPKD, this type of kidney disease is associated with various factors that place children with ARPKD at risk for neurological and neuropsychological impairment. In particular, this mini review highlighted several of the conditions that can accompany the kidney disease including hypertension, liver disease, and chronic lung disease. Additional complexities that can affect the cognitive and psychosocial functioning of this population include the need for an eventual kidney transplant, possible dual transplantation of the kidneys and liver, and associated medical needs, such as dialysis early in life for some of the more severely involved infants. Importantly, these factors are present during a critical period of brain development and not only contribute to proximal neurocognitive problems but also to possible distal, downstream neurocognitive problems over the course of development. Specific challenges and directions for the field of pediatric nephrology remain present for ARPKD, particularly with respect to advancing best clinical practices for professionals working with these children and guiding potential research directions for this rare condition.

## Author Contributions

SH conceptualized and drafted the entire manuscript.

## Conflict of Interest Statement

The author declares that the research was conducted in the absence of any commercial or financial relationships that could be construed as a potential conflict of interest.

## References

[1] Dell KM. The spectrum of polycystic kidney disease in children. Adv Chronic Kidney Dis (2011) 18:339–47. 10.1053/j.ackd.2011.05.001 21896375 PMC3168776

[2] Patil A Sweeney WE Avner ED Pan C Childhood polycystic kidney disease. In: Li X, editor. Polycystic Kidney Disease. Brisbane, Australia: Codon Publications (2015). p. 1–25. 27512782

[3] Bergmann C. ARPKD and early manifestations of ADPKD: the original polycystic kidney disease and phenocopies. Pediatr Nephrol (2015) 30:15–30. 10.1007/s00467-013-2706-2 24584572 PMC4240914

[4] Rajanna DK Reddy A Srinivas NS Aneja A. Autosomal recessive poly cystic kidney disease: antenatal diagnosis and histopathological correlation. J Clin Imaging Sci (2013) 3:13. 10.4103/2156-7514.109733 23814685 PMC3690676

[5] Roy S Dillon MJ Trompeter RS Barratt TM. Autosomal recessive polycystic kidney disease: long-term outcome of neonatal survivors. Pediatr Nephrol (1997) 11:302–6. 10.1007/s004670050281 9203177

[6] Khan K Schwarzenberg SJ Sharp HL Matas AJ Chavers BM. Morbidity from congenital hepatic fibrosis after renal transplantation for autosomal recessive polycystic kidney disease. Am J Transplant (2002) 2:360–5. 10.1034/j.1600-6143.2002.20412.x 12118859

[7] Dell KM Matheson M Hartung EA Warady BA Furth SL Chronic Kidney Disease in Children (CKiD) Study. Kidney disease progression in autosomal recessive polycystic kidney disease. J Pediatr (2016) 171:196–201. 10.1016/j.jpeds.2015.12.079 26831744 PMC5349855

[8] Bergmann C Senderek J Windelen E Kipper F Middeldorf I Schneider F Clinical consequences of PKHD1 mutations in 164 patients with autosomal-recessive polycystic kidney disease (ARPKD). Kidney Int (2005) 67:829–48. 10.1111/j.1523-1755.2005.00148.x 15698423

[9] Guay-Woodford LM Desmond RA. Autosomal recessive polycystic kidney disease: the clinical experience in North America. Pediatrics (2003) 111:1072–80. 10.1542/peds.111.5.1072 12728091

[10] Bergmann C Ciliopathies. Eur J Pediatr (2012) 171:1285–300. 10.1007/s00431-011-1553-z 21898032 PMC3419833

[11] Guay-Woodford LM Galliani CA Musulman-Mroczek E Spear GS Guillot AP Bernstein J. Diffuse renal cystic disease in children: morphologic and genetic correlations. Pediatr Nephrol (1998) 12:173–82. 10.1007/s004670050431 9630032

[12] Lacerda G Krummel T Hirsch E. Neurologic presentations of renal diseases. Neurol Clin (2010) 28:45–59. 10.1016/j.ncl.2009.09.003 19932375

[13] Zeir MG The suddenly speechless florist on chronic dialysis: the unexpected threats of a flower shop? Nephrol Dial Transplant (2006) 21:223–5. 10.1093/ndt/gfh990 16280376

[14] Yardimci N Colak T Sevmis S Benli S Zileli T Haberal M. Neurologic complications after renal transplant. Exp Clin Transplant (2008) 6:224–8. 18954301

[15] Hooper SR Gerson AC Neurocognitive functioning and related outcomes in pediatric renal disease. In: Baron IS Rey-Casserly C, editors. Lifespan Neuropsychology. New York: Oxford University Press (2013). p. 158–76.

[16] Hooper SR Gerson AC Butler RW Gipson DS Mendley SR Lande MB Neurocognitive functioning of children and adolescents with mild-to-moderate chronic kidney disease. Clin J Am Soc Nephrol (2011) 6(8):1824–30. 10.2215/CJN.09751110 21737850 PMC3156421

[17] Greenbaum LA Warady BA Furth SL Current advances in chronic kidney disease in children: growth, cardiovascular, and neurocognitive risk factors. Semin Nephrol (2009) 29(4):425–34. 10.1016/j.semnephrol.2009.03.017 19615563 PMC2765584

[18] Adams HR Szilagyi PG Gebhardt L Lande MB. Learning and attention problems among children with pediatric primary hypertension. Pediatrics (2010) 126:1425–9. 10.1542/peds.2010-1899 21059718 PMC5578423

[19] Kupferman JC Lande MB Adams HR Pavlakis SG. Primary hypertension and neurocognitive and executive functioning in school-age children. Pediatr Nephrol (2013) 28:401–8. 10.1007/s00467-012-2215-8 22692504 PMC3666570

[20] Lande MB Gerson AC Hooper SR Cox C Matheson M Mendley SR Casual blood pressure and neurocognitive function in children with chronic kidney disease: a report of the children with chronic kidney disease cohort study. Clin J Am Soc Nephrol (2011) 6:1831–7. 10.2215/CJN.00810111 21700829 PMC3156422

[21] Lande MB Kupferman JC Adams HR. Neurocognitive alterations in hypertensive children and adolescents. J Clin Hypertens (2012) 14:353–9. 10.1111/j.1751-7176.2012.00661.x 22672088 PMC3375877

[22] Lande MB Batisky DL Kupferman JC Samuels J Hooper SR Falkner B Neurocognitive function in children with primary hypertension. J Pediatr (2017) 180:148–55. 10.1016/j.jpeds.2016.08.076 27692987 PMC5183510

[23] Lande MB Mendley SR Matheson M Shinnar S Gerson AC Association of blood pressure variability and neurocognition in children with chronic kidney disease. Pediatr Nephrol (2016) 31:2137–44. 10.1007/s00467-016-3425-2 27263021 PMC5042825

[24] Jennings JR. Autoregulation of blood pressure and thought: preliminary results of an application of brain imaging to psychosomatic medicine. Psychosom Med (2003) 65:384–95. 10.1097/01.PSY.0000062531.75102.25 12764211

[25] Lande MB Adams H Falkner B Waldstein SR Schwartz GJ Szilagyi PG Parental assessment of executive function and internalizing and externalizing behavior in primary hypertension after anti-hypertensive therapy. J Pediatr (2010) 157:114–9. 10.1016/j.jpeds.2009.12.053 20227722 PMC2904985

[26] Srinath A Shneider BL. Congenital hepatic fibrosis and autosomal recessive polycystic kidney disease. J Pediatr Gastroenterol Nutr (2012) 54:580–7. 10.1097/MPG.0b013e31824711b7 22197937 PMC4369775

[27] Tsimaratos M Cloarec S Roquelaure B Retornaz K Picon G Chabrol B Chronic renal failure and portal hypertension – is portosystemic shunt indicated? Pediatr Nephrol (2000) 14:856–8. 10.1007/s004679900268 10955945

[28] Kappus MR Bajaj JS. Covert hepatic encephalopathy: not as minimal as you might think. Clin Gastroenterol Hepatol (2012) 10:1208–19. 10.1016/j.cgh.2012.05.026 22728384

[29] Alonso EM. Quality of life for pediatric liver recipients. Liver Transpl (2009) 15:S57–62. 10.1002/lt.21904 19876952

[30] Sanchez C Eymann A De Cunto C D’Agostino D. Quality of life in pediatric liver transplantation in a single-center in South America. Pediatr Transplant (2010) 14:332–6. 10.1111/j.1399-3046.2009.01225.x 19735435

[31] Denny B Beyerle K Kienhuis M Cora A Gavidia-Payne S Hardikar W. New insights into family functioning and quality of life after pediatric liver transplantation. Pediatr Transplant (2012) 16:711–5. 10.1111/j.1399-3046.2012.01738.x 22775776

[32] Wu YP Aylward BS Steele RG. Associations between internalizing symptoms and trajectories of medication adherence among pediatric renal and liver transplant recipients. J Pediatr Psychol (2010) 35:1016–27. 10.1093/jpepsy/jsq014 20231258

[33] Krull K Fuchs C Yurk H Boone P Alonso E. Neurocognitive outcome in pediatric liver transplant recipients. Pediatr Transplant (2003) 7:111–8. 10.1034/j.1399-3046.2003.00026.x 12654051

[34] Laughon M O’Shea MT Allred EN Bose C Kuban K Van Marter LJ Chronic lung disease and developmental delay at 2 years of age in children born before 28 weeks gestation. Pediatrics (2009) 124:637–48. 10.1542/peds.2008-2874 19620203 PMC2799188

[35] Farel AM Hooper SR Teplin SW Henry MM Kraybill EN Very low birthweight infants at seven years: an assessment of the health and neurodevelopmental risk conveyed by chronic lung disease. J Learn Disabil (1998) 31:118–26. 10.1177/002221949803100202 9529782

[36] Short EJ Klein NK Lewis BA Fulton S Eisengart S Kercsmar C Cognitive and academic consequences of bronchopulmonary dysplasia and very low birth weight: 8-year-old outcomes. Pediatrics (2003) 112:e359–66. 10.1542/peds.112.5.e359 14595077 PMC10222516

[37] Carter SA Kitching AR Johnstone LM. Four pediatric patients with autosomal recessive polycystic kidney disease developed new-onset diabetes after renal transplantation. Pediatr Transplant (2014) 18:698–705. 10.1111/petr.12332 25118046

[38] Liu SP Ding J Wang F Zhang YQ Ye JT. Clinical characteristics and mutation analysis of three Chinese children with autosomal recessive polycystic kidney disease. World J Pediatr (2014) 10:271–4. 10.1007/s12519-014-0503-z 25124979

[39] Hartung EA Matheson M Lande MB Dell KM Guay-Woodford LM Gerson AC Neurocognition in children with autosomal recessive polycystic kidney disease in the CKiD cohort study. Pediatr Nephrol (2014) 29:1957–65. 10.1007/s00467-014-2816-5 24828609 PMC4167962

[40] Guay-Woodford LM Bissler JJ Braun MC Bockenhauer D Cadnapaphornchai MA Dell KM Consensus expert recommendations for the diagnosis and management of autosomal recessive polycystic kidney disease: report of an international conference. J Pediatr (2014) 165:611–7. 10.1016/j.jpeds.2014.06.015 25015577 PMC4723266

[41] Guay-Woodford LM. Autosomal recessive polycystic kidney disease: the prototype of the hepato-renal fibrocystic diseases. J Pediatr Genet (2014) 3:89–101. 25632369 10.3233/PGE-14092 PMC4306463

[42] Hoyer PF. Clinical manifestations of autosomal recessive polycystic kidney disease. Curr Opin Pediatr (2015) 27:186–92. 10.1097/MOP.0000000000000196 25689455

[43] Miller CK Burklow KA Santoro K Kirby E Mason D Rudolph CD An interdisciplinary team approach to the management of pediatric feeding and swallowing disorders. Child Health Care (2010) 3:201–18.

[44] Ebner K Feldkoetter M Ariceta G Bergmann C Buettner R Doyon A Rationale, design, and objectives of ARegPKD, a European ARPKD registry study. BMC Nephrol (2015) 16:22. 10.1186/s12882-015-0002-z 25886171 PMC4359504

